# Soil carbon formation is promoted by saturation deficit and existing mineral-associated carbon, not by microbial carbon-use efficiency

**DOI:** 10.1126/sciadv.adv9482

**Published:** 2025-06-13

**Authors:** Alison E. King, Noah W. Sokol

**Affiliations:** ^1^ Department of Soil and Crop Sciences, Colorado State University, Fort Collins, CO, USA.; ^2^ School of Food and Agriculture, University of Maine, Orono, ME, USA.; ^3^ Physical & Life Science Directorate, Lawrence Livermore National Laboratory, Livermore, CA, USA.

## Abstract

Mineral-associated organic carbon (MAOC) is the largest terrestrial pool of organic carbon, yet controls on its formation remain unresolved. Existing MAOC is thought to preclude additional C storage on minerals, but this perspective is difficult to reconcile with observations that MAOC stacks in multilayers, suggesting that existing MAOC could promote greater C retention. Here, in a manipulative experiment using 118 soils from 15 agricultural sites across the United States, we show that MAOC formation is promoted by both existing MAOC and its counterpart—MAOC saturation deficit. The positive effect of existing MAOC on the formation of new MAOC persists after accounting for soil physicochemical properties that covary with MAOC. In contrast with current theory, we found that MAOC formation was not clearly influenced by microbial carbon-use efficiency (CUE). Our findings demonstrate that existing MAOC and saturation deficit, not microbial CUE, are key to determining new MAOC formation in agricultural soils.

## Teaser

Manipulative study on 118 soils shows that MAOC formation increases with existing MAOC concentration and saturation deficit.

## INTRODUCTION

Mineral-associated organic carbon (MAOC) is Earth’s largest pool of terrestrial organic carbon (C) (*1*) and makes up ~70 to 80% of total soil organic carbon (SOC) across Earth’s agricultural soils (*1*, *2*). Managing agricultural soils for enhanced SOC storage to mitigate climate change (*3*) offers a win-win for soil health as greater SOC is associated with higher and more stable crop yields (*4*, *5*). Despite these benefits, considerable uncertainty remains as to the environmental factors that moderate formation efficiency of MAOC from plant C inputs. Both mineralogical and microbial properties of soil have been positioned as critical for the formation of MAOC (*6*, *7*), although these sets of properties have rarely been tested in tandem to determine their relative influence on MAOC formation.

Early reports revealed that MAOC contents increase with the soil silt + clay fraction (*8*, *9*). This observation, together with an historical assumption that the accumulation of MAOC occurs in a monolayer onto soil mineral surfaces (*10*), has led to a framework in which the availability of bare mineral surfaces determines how much new MAOC can be formed (*11*–*13*). To quantify the remaining storage potential of a soil to store MAOC, known as the MAOC saturation deficit, researchers have often described a maximum MAOC saturation from the increase in MAOC concentration with silt + clay contents (*14*–*16*) and found the difference between existing MAOC and expected MAOC saturation. Because soils that are high in iron and aluminum oxides (Fe_O_ and Al_O_) and exchangeable calcium (Ca_ex_) also contain large MAOC pools (*17*, *18*), this saturation line is sometimes calculated separately for soils grouped according to their mineralogical properties (*15*, *19*). A central pillar of this approach to MAOC storage capacity, which is prevalent in process-based and conceptual models of soil biogeochemistry, is that the potential for MAOC formation diminishes with increasing MAOC concentrations (at a given texture and mineralogy) because a greater proportion of mineral binding sites are putatively already occupied by organic carbon (OC).

The idea that OC forms a monolayer on available mineral binding sites (*10*) is still invoked, although the occurrence of purely monolayer MAOC accumulation has been refuted by reports of multilayer stacking of MAOC—in which OC binds to other OC, forming a “stack” of OC bound to a mineral at its base (*20*–*22*). Given this multilayer stacking of MAOC, it seems unlikely that the availability of bare mineral surfaces alone controls the formation of new MAOC. The observations of multilayer MAOC stacking imply that existing MAOC should promote more new MAOC formation. However, because researchers have relied heavily on using bare minerals to study MAOC formation instead of whole soils (*17*, *23*, *24*), the influence of existing MAOC on new MAOC formation has largely been untested. No manipulative experiments to date have tested the possibility that existing MAOC and MAOC saturation deficit could simultaneously regulate MAOC formation.

Alongside mineralogical limitations on MAOC storage, microbial carbon-use efficiency (CUE) has been theorized to be a strong driver of MAOC formation (*6*). Namely, if new inputs of organic C are microbially processed before entering the MAOC pool (*25*), and if soils have a MAOC saturation deficit (*7*), then low microbial CUE could limit MAOC formation. Although some observational studies support a positive relationship between CUE and SOC (*26*, *27*), few manipulative experiments have sought to isolate a causal pathway for the role of CUE in controlling MAOC formation efficiency. The very few studies that have tested the relationship between microbial CUE and MAOC have found both negative (*28*, *29*) and positive (*30*) relationships. Thus far, however, few studies have tested the relative role of CUE versus mineralogical variables, especially saturation deficit. Moreover, no manipulative experiments to date have examined how differences in the rate of C input shape how microbial CUE influences MAOC formation. Higher rates of C inputs have been associated with lower MAOC formation efficiencies (*31*), which creates an opportunity to test whether these lower formation efficiencies are explained by lower microbial CUE.

Here, we tested in a ^13^C isotope tracing experiment how the interplay between existing MAOC concentration, MAOC saturation deficit, and microbial CUE affected the formation of new MAOC. We selected a range of 118 plots across 15 agricultural sites in the continental US that varied in a wide range of properties, including their existing MAOC concentration, as well as in their MAOC saturation status and soil physicochemical properties (*18*) (fig. S1 and table S1). These soils were then subjected to two distinct C addition treatments in laboratory microcosms: a regular input of ^13^C-DOC at fixed location (generating a high C input treatment) versus a dispersed, irregular application of the same ^13^C-DOC and at only half the concentration, generating a low C input treatment. These treatments simulated how C enters into some regions of the soil as a more concentrated input (e.g., the area directly around the living root—the rhizosphere) versus other regions of the soil as a more diffuse input, spread across a greater area (e.g., the bulk soil) (*31*). Treatments and observations were carried out over a 6-month period, commensurate with the growth cycle of an agricultural cover crop, for a total of 25 ^13^C-DOC additions. Regular trace gas measurements throughout the incubation allowed assessment of the proportion of ^13^C-DOC respired as CO_2_ during the initial phase of rapid microbial consumption of ^13^C-DOC; formation of MAOC from the ^13^C-DOC and microbial CUE were assessed at the termination of the incubation. We asked: (i) Do soils with greater existing MAOC concentrations support greater or lesser formation of new MAOC? (ii) Do soils farther from their MAOC saturation deficit support greater MAOC formation? and (iii) Does microbial CUE influence the formation efficiency of MAOC under varying input levels of C input?

## RESULTS AND DISCUSSION

### Formation of MAOC is regulated by existing MAOC concentration and saturation deficit

**Fig. 1.**
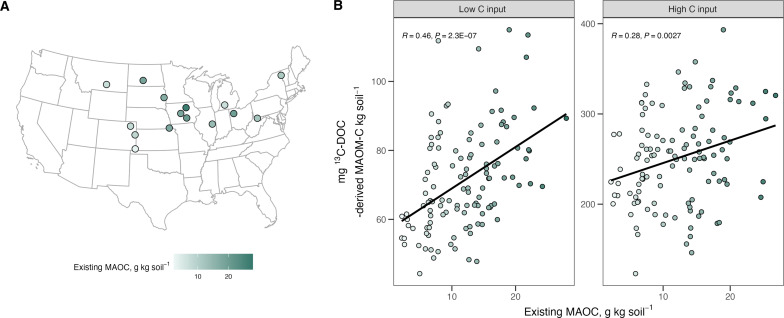
Patterns in MAOC across scales. (**A**) Map of study sites in the United States. At each site, 6 to 10 subplots were sampled and incubated with 25 weekly applications of ^13^C-DOC under two C input treatments (low, *n* = 116; high, *n* = 115), after which (**B**) formation of new MAOC was quantified, here shown as a function of existing MAOC.

Across 15 different agricultural sites and 118 plots, we found that there was a strong positive relationship between the amount of new MAOC formed and existing MAOC concentration (Fig. 1, *R* = 0.28 to 0.46; *P* < 0.01). Our results are consistent with isotope tracing work in the field, where existing concentrations of total SOC promote formation of new MAOC (*32*). Our results are also consistent with a previous isotope tracing work under laboratory conditions where, in two contrasting soils, existing MAOC promoted formation of new MAOC (*20*). Our results provide more substantive evidence for the role of existing MAOC in promoting new MAOC formation, by showing this relationship through a controlled experiment and across a much wider range of soils.

**Fig. 2.**
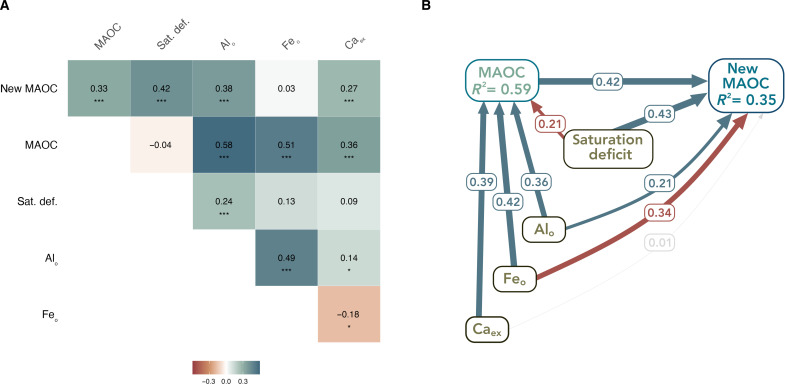
Relationships between soil properties, including existing MAOC, and the formation of new MAOC. (**A**) Multilevel Pearson’s correlation coefficients for relationships between formation of new MAOC (assessed as retention of a ^13^C-DOC label in MAOC, mg ^13^C-DOC-derived MAOC kg soil^−1^) and existing MAOC (MAOC, g C kg soil^−1^), oxalate-extractable aluminum (Al_O_, g kg soil^−1^), oxalate-extractable iron (Fe_O_, g kg soil^−1^), exchangeable calcium (Ca_ex_, cmol kg soil^−1^), and saturation deficit (sat.def.; g C kg soil^−1^), across both levels of C input addition. (**B**) Structural equation model, in which standardized path coefficients were averaged across low and high C input treatments after multilevel SEM testing indicated no significant difference between a constrained model (regression coefficients forced to be the same between treatments) and an unconstrained model (regression coefficients could vary between treatments). Blue paths represent significant positive relationships (*P* < 0.05); red paths represent significant negative relationships (*P* < 0.05); gray path represents nonsignificant relationships. Both models have comparative fit index (CFI) > 0.95 and root mean square error of approximation (RMSEA) < 0.08. For both high and low C input treatments, *n* = 107 after uniform outlier cleaning procedure (see the section “Statistical analyses”).

The formation of new MAOC also increased with MAOC saturation deficit (Fig. 2, A and B). Although a role for saturation deficit in regulating formation of new MAOC is widely expected, a quantification of MAOC saturation deficit as a driver of MAOC formation has not been clearly demonstrated. Our results indicate that, although most soils were far from MAOC saturation, as is typical in agricultural soils (*2*), MAOC saturation nevertheless influenced MAOC formation across high versus low C input treatments. Although saturation status has been theorized to influence the formation of particulate organic carbon (POC) as well (*7*), in this study, the formation of new POC from the ^13^C-DOC input was two orders of magnitude lower than formation of new MAOC (fig. S2), as expected with the use of this nonstructural ^13^C-DOC label, since it is structural inputs that primarily enter the POC pool (*33*). For the formation of new MAOC, our results suggest a dual role of both mineral surfaces and existing MAOC as a base for formation of new MAOC.

To accurately assess the relationship between existing MAOC concentrations and new MAOC formation, it is important to also consider the soil physicochemical properties that covary with MAOC. For instance, soils with greater quantities of Al_O_, Fe_O_, and Ca_ex_ typically contain higher MAOC or SOC levels (*18*, *34*, *35*). Thus, in our soils, it is possible that soil physicochemical properties (such as concentrations of Al_O_, Fe_O_, Ca_ex_, or the percent of silt and clay) may have also contributed to new MAOC formation to an equal or greater extent than existing MAOC concentration. To test this possibility, we constructed a structural equation model to test causal pathways influencing formation of new MAOC. In this model, soil physicochemical properties were given an opportunity to increase MAOC, whereas MAOC saturation deficit reduced existing MAOC, and all properties could influence formation of new MAOC. This choice of causal pathway was informed by model fit indices, which indicated poor model fit when MAOC influenced saturation deficit. Although silt + clay concentration did influence saturation deficit in these soils, it was removed from the structural equation models due to high collinearity with MAOC saturation deficit, which would have caused incorrect estimation of path coefficients. Substituting silt + clay for saturation deficit resulted in reduced explanatory power over new MAOC formation (fig. S3). Structural equation modeling revealed that, after the influence of other soil physiochemical properties on new MAOC had been accounted for, across both high and low C input treatments, there was still a significant effect of existing MAOC on new MAOC formation (Fig. 2B).

As with soil minerals, OC molecules have the capacity to form associations with other OC molecules due to their surface charges—known as “organo-organic interactions” (*21*, *36*). Because of these organo-organic interactions and multilayer stacking of OC, a greater amount of existing MAOC reasonably leads to greater new MAOC formation. We also observed the C:N ratio of newly-formed MAOC increasing with soil Ca_ex_ (fig. S4). This relationship could be because soils with greater matrix capacity to stabilize MAOC, e.g., through cation bridging with Ca_ex_, formed MAOC via direct sorption of DOC in organo-mineral or organo-organo interactions, rather than through a microbial formation pathway. Calcium may have also been associated with greater capacity for aggregate protection of DOC (*35*). As a result, the newly formed MAOC may be less microbially processed in soils higher in Ca_ex_ as its C:N ratio is closer to that of the added ^13^C-DOC.

### Formation efficiency of MAOC not explained by microbial CUE

**Fig. 3.**
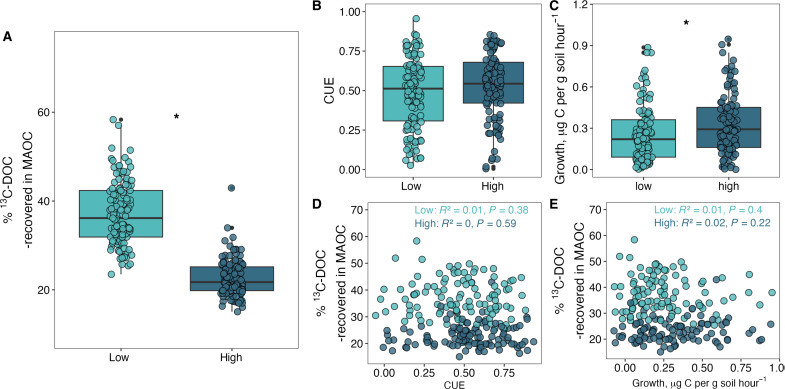
Microbial CUE and growth rate do not explain differences in formation efficiency of MAOC between or within C input treatments. (**A**) Differences in formation efficiency of MAOC, assessed as % ^13^C-DOC recovered in MAOC (low, *n* = 115; high, *n* = 118), between C input treatments. (**B**) Microbial CUE (low, *n* = 111; high, *n* = 110) and (**C**) microbial growth rate (low, *n* = 108; high, *n* = 99). An asterisk between C input treatment boxplots indicates difference between treatments at *P* < 0.05. (**D**) Formation efficiency of MAOC in both C input treatments as a function of CUE and (**E**) formation efficiency of MAOC in both C input treatments as a function of microbial growth rate.

As hypothesized, a higher rate of C input led to lower proportion of ^13^C-DOC that was retained as MAOC (Fig. 3A). In the low C input treatment, 37% of ^13^C-DOC applied was retained as MAOC, whereas only 23% of ^13^C-DOC applied was retained under the higher rate of input. Microbial CUE likely did not explain these differences because CUE [measured via the ^18^O-H_2_O method (*37*)] was not significantly different between these treatments (*P* = 0.14; Fig. 3B). Microbial community-level growth rates also did not explain differences in formation efficiency, as growth rates increased slightly in the high C input treatment (*P* < 0.05; Fig. 3C) and did not explain formation efficiency differences within treatments (Fig. 3E). Microbial CUE and growth were poor predictors of absolute MAOC formation (fig. S5, *R*^2^ < 0.05). It is possible that other differences in microbial physiology influenced differential MAOC formation efficiencies in the high versus low input treatments (such as extracellular enzyme activity or extracellular polymeric substances) (*29*), but these differences were not related to microbial CUE or growth rate—two of the most commonly measured microbial traits related to the microbial formation pathway of MAOC (*1*, *6*, *29*, *38*).

The low C input treatment may have stabilized MAOC with greater efficiency than the high C input treatment if it favored a more efficient, direct sorption pathway, compared to a less efficient, microbial formation pathway, where C can be lost via microbial respiration. We found that the low C input treatment was associated with reduced microbial uptake of the ^13^C-DOC input (fig. S6), as quantified by ^13^C-DOC respiration, and more was retained as MAOC. Specifically, in this experiment and in other experiments tracing isotopically-enriched DOC, respiration of the DOC follows a biphasic pattern: initial rapid respiration of the DOC, followed by slow respiration (*39*) (fig. S7). In the initial phase, there may have been greater spatial proximity of microbes to ^13^C-DOC in the high C input treatment due to more concentrated ^13^C-DOC g soil^−1^, or greater microbial biomass (fig. S8), which could facilitate greater microbial uptake (*40*). Such data support the idea that the low C input treatment may have facilitated greater direct sorption versus microbial uptake of ^13^C-DOC. Lower microbial uptake of ^13^C-DOC in the low C input treatment is also supported by slightly higher and more uniform C:N ratio of newly formed MAOC in the low versus high C input treatments [13.1 versus 12.7 C:N ratio; interquartile range (IQR) 1.93 versus 1.04; fig. S9, *P* = 0.047], indicating that MAOC in the low C input treatment had a higher ratio of unprocessed DOC to microbial necromass and a less consistent degree of microbial processing (*41*, *42*). Lower microbial uptake of ^13^C-DOC in the low versus high C input treatment is also supported by lower proportional losses of ^13^C-DOC in the initial phase of respiration in the low C input treatment (fig. S6, *P* < 0.001). In this experiment, the high C input treatment lost 54% of applied ^13^C-DOC in initial respiration phases, compared to 37% in the low C input treatments, which may have also been caused by lower concentrations of DOC in soil solution, or other aspects of microbial physiology not captured by the CUE assay. Overall, variability in microbial uptake between treatments and systems (*41*) underscores a broad challenge of linking measures of microbial CUE to MAOC formation.

**Fig. 4.**
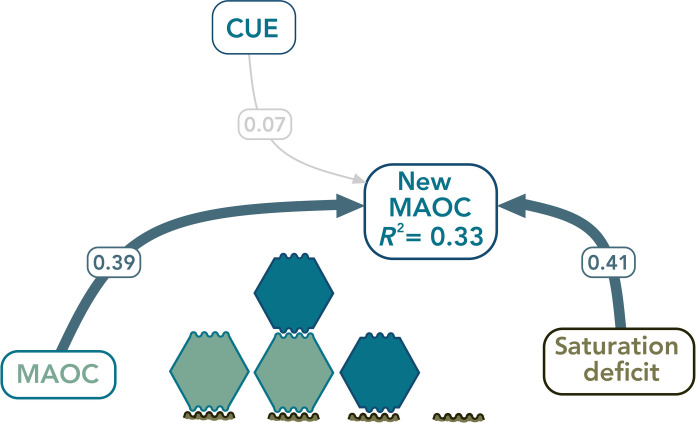
Simplified structural equation model showing controls on new MAOC formation. Predictor variables shown include saturation deficit (g C kg soil^−1^) of MAOC, microbial CUE, and existing MAOC (MAOC). Standardized path coefficients were averaged across low and high C input treatments after multilevel SEM testing indicated no significant difference between a constrained model (regression coefficients forced to be the same between treatments) and an unconstrained model (regression coefficients could vary) (*n* = 115). Blue paths represent significant positive relationships (*P* < 0.05); gray path represents nonsignificant relationship. The model has CFI > 0.95 and RMSEA < 0.08.

Microbial CUE was highly variable within high and low C input treatments (Fig. 3B), as was microbial community-level growth rate (Fig. 3E), neither of which explained differences in MAOC formation efficiency (*R*^2^ < 0.02; *P* > 0.22) or absolute MAOC formation (fig. S5). Although the substrate-independent nature of the ^18^O-CUE approach is often highlighted as an advantage of this CUE method (*37*), this feature may also be a limitation when examining the fate of a specific substrate. Microbial growth efficiencies can vary between different substrates (*43*), such as between the existing SOC and newly added DOC. Consequently, if the aim is to track the fate of a DOC label, then the substrate-independent nature of the ^18^O-CUE approach might obscure the microbial processing of the label with the simultaneous consumption of existing SOC. Our results supply an important rationale to reexamine the assumption that substrate-independent tests of CUE are more reliable than substrate-specific tests. Revisiting substrate-specific measures of CUE—or other aspects of microbial metabolism—will be particularly relevant if studies are to be manipulative (i.e., tracking specific isotopically labeled compounds with microbial metabolism as putative control on MAOC formation) rather than observational (i.e., measuring microbial metabolism and its correlation with MAOC pools). In this application of a widely used CUE measurement to test its capacity to predict MAOC formation, we found that features of the soil matrix—existing MAOC and MAOC saturation deficit—emerged as stronger determinants of MAOC formation than microbial CUE (Fig. 4).

### Role of MAOC and microbial traits in MAOC accumulation

Understanding the primary controls on MAOC formation is critical for determining how much C can be accrued in soils. To date, much attention has focused on the availability of mineral surfaces in soil that can adsorb OC, as well as the efficiency of the microbial community (often measured as microbial CUE) on controlling the formation of new MAOC (*27*, *44*). Here, by assessing formation of new MAOC across soils ranging in both MAOC and saturation deficit and then assaying microbial CUE, we demonstrate that both existing MAOC concentrations and MAOC saturation deficit increase new MAOC formation. In contrast with current theory, we found that microbial CUE did not clearly influence MAOC formation. Our results suggest that the concentration or rate of DOC inputs itself may be an important feature that moderates MAOC formation efficiency by controlling microbial uptake versus direct sorption.

The observation that greater existing MAOC concentrations promote greater new MAOC formation opens key questions about how soils can optimally accrue MAOC and how persistent new MAOC will be over time. If existing MAOC stocks do not become more vulnerable to destabilization as their size increases, or if this effect is minimal, then soils with the greatest MAOC stocks hold substantial potential to store more C. However, a simple positive relationship between MAOC stocks and net MAOC formation and persistence is not clearly supported by other lines of evidence, such as diminishing returns in SOC accumulation across a gradient of C inputs (*45*). It is likely that turnover of a portion of the MAOC pool increases with greater accrual of MAOC, such that the formation of new MAOC is partially or completely balanced out the by destabilization of existing MAOC. This possibility is consistent with the expectation that more “stacked” MAOC is more vulnerable to destabilization (*36*) and underscores emerging understanding of MAOC as a fraction with heterogeneous turnover times (*46*)—i.e., some less vulnerable MAOC at the base of MAOC stacks and some rapidly cycling MAOC that attracts formation of new MAOC and is likely to be destabilized through a priming process (*47*). A critical next step will be to better understand how durable new MAOC is across soils with a range of different MAOC concentrations and different proximities to a theorized C saturation point.

Future work should also focus on untangling the mechanisms controlling OC sorption to both vacant and occupied mineral surfaces. As existing MAOC can promote MAOC formation, as we report here, mechanisms other than the absence of vacant mineral surfaces are needed to explain the saturation, or nonaccumulation, of MAOC over time. Continued reliance on bare, or pristine, minerals to study MAOC dynamics is likely to yield an incomplete picture of the drivers of MAOC cycling in natural soils, given the common presence of OC coatings on soil minerals. Understanding the extent to which the findings presented here generalize across forest and grassland ecosystems and under in situ temperature and moisture conditions will also be critical for predicting the accrual of MAOC across different environmental contexts. It will also be important to refine CUE measurement approaches and test a wider suite of microbial traits, beyond CUE and growth rate (such as the amount and chemistry of extracellular polymeric substances) (*48*). Together, these research avenues will clarify the features of soil microbial communities, the soil matrix, and existing OC that constrain net MAOC accumulation, in order to optimally manage soils for C storage.

## MATERIALS AND METHODS

### Site selection and soil sampling

Soils from 15 agricultural sites across the United States (*18*) were sampled in fall 2020 from the top 20 cm. Soils were transported to Colorado State University campus on ice and transferred to 4°C walk-in upon arrival. All soils were sieved to 8 mm while fresh, removing only roots and rocks remaining on sieve surface. Air-dried soils were root picked for 2 min for every 100 dry grams of soil. Soils were stored air-dried until the incubation experiment was initiated in March 2021.

### Soil physicochemical properties

A suite of soil physicochemical properties was assessed on baseline soils only and previously reported (*18*); a summary table of soil properties is provided in table S1, and the full dataset is available in a Figshare repository (https://doi.org/10.6084/m9.figshare.25035818). Among these properties, only soil pH and soil C were assessed again on incubated soils. For both baseline and postincubation measurements, soil pH was assessed in a slurry of 1:1 soil:water by mass after 10 min of rest. On baseline soils, soil texture was assessed using the hydrometer method to determine clay content, and mass recovery of sand particles (>53 μm) was used to determine sand content. Soil exchangeable cations were extracted in ammonium acetate (*49*). Briefly, ~2.5 g of 2-mm sieved, air-dried soil was shaken for 30 min with 25 ml of 1 M ammonium acetate at pH 7 and then filtered through a Whatman #1 filter paper (nominal pore size = 11 μm) and analyzed via inductively coupled plasma optical emission spectrometer (ICP-OES; Optima 7300 DV, PerkinElmer, Waltham, MA, USA) for Ca^2+^. Soil Fe and Al were extracted via the acid ammonium oxalate method following (*50*), which estimates Fe and Al in amorphous phases (referred to as Fe_O_ and Al_O_). For the acid ammonium oxalate extraction, ~0.5 g of ground, air-dried soil was mixed with 30 ml of 0.175 M ammonium oxalate at pH 3 and shaken for 2 hours in the dark and then filtered through a Whatman #1 filter paper. Calcareous soils were identified by reaction with HCl, as described below, and their carbonates removed before the acid ammonium oxalate extraction by reacting the sample with 30 ml of 1 M ammonium acetate at pH 5.5. Ammonium oxalate extracts were also run via ICP-OES. All analyses performed on air-dried soils are reported in oven-dry equivalents. Soil carbon saturation deficit was estimated following Georgiou *et al*. (*15*), as previously reported for these soils (*2*), and described below.

### Preparation of dual-labeled DOC

Ryegrass was produced in a dual isotope labeling chamber (*51*), in which ^13^C-CO_2_ and ^15^N fertilizer were continuously supplied to create uniformly labeled tissues, where ^13^C-enriched was ~4 atomic % (at %). Although formation of MAOC is the primary focus of this study, we leveraged the ^15^N label to quantify the C:N ratio of newly formed MAOC. To prepare a DOC solution representative of soluble root inputs, both shoots and roots were clipped to ~2 cm and extracted in hot water for 3 hours at 100°C, with a ratio of 0.7 g dry litter to 35 ml of deionized (DI) water (*52*). Shoot inputs were included to generate sufficient plant mass for C additions throughout the entire experiment. This solution was filtered through sequentially finer filters (20, 0.22, and lastly 0.1 μm) to remove solids that could have clogged rhizons, which had a 0.15-μm pore size. Two DOC solutions were prepared in more and less concentrated forms to correspond to high or low C input treatments: DOC for high C input microcosms contained 1.37 mg C ml solution^−1^ and DOC for low C input microcosms contained 0.68 mg C ml solution^−1^ (analyzed for C concentration on a Shimadzu–total organic carbon analyzer for nonpurgeable organic carbon). The DOC solution was freeze-dried and ground in a mortar and pestle prior to running on via Elemental Analyzer–Isotope Ratio Mass Spectrometry (EA-IRMS; vario ISOTOPE cube, Elementar, Langenselbold, Germany), which retuned a δ^13^C of 2958‰ (per mil) and C:N ratio of 34. Both low and high DOC solutions were partitioned into containers portioned for a single day’s application of DOC, frozen, and thawed on the day of use to ensure consistent DOC quality throughout the experiment.

### Soil microcosms

Microcosms were constructed from sheets of 1/4-inch (6.35-mm) hard acrylic with neoprene sides, with 100-cm^3^ internal volume. To determine exact soil mass for each microcosm, soils from each plot were first assessed for their water holding capacity (WHC; g water retained/g soil after 2 hours allowed to drain while covered). Given the highly variable WHC between soils (0.42 to 0.76 g water/g soil), and the need to maintain soils at a moderate level of soil water (45% WHC) to maintain optimal microbial activity, soils could not be packed into microcosms to a consistent bulk density across soils. Microcosms were instead standardized to a consistent total mass of soil + water = 175 g. Bulk density therefore ranged from 1.30 to 1.47 g/cm^3^, representative of available information on bulk density from these agricultural soils. Half of all microcosms were designated as low C input, whereas the other half were designated as high C input. In high C input microcosms, microrhizon samplers, 10 cm in rhizon length, 2.5 mm in diameter, and 0.15-μm pores (SoilMoisture Equipment Corp., Goleta, CA), were inserted midway into the microcosm to deliver ^13^C-DOC to a consistent location. Microcosms were maintained at 45% WHC through biweekly additions of DI water. Although fluctuations in water content of microcosms were small, additions of DI water occurred at least 48 hours prior to any gas sampling to avoid potential CO_2_ flushes associated with wetting.

### Addition of ^13^C-DOC to soil microcosms

^13^C-DOC, described above, was applied weekly to each microcosm for 25 weeks. Before the first ^13^C-DOC addition, soils were incubated at 45% WHC and 25°C for 1 week to enable determination of natural abundance δ^13^C-CO_2_ from each microcosm (described below). Natural abundance δ^13^C-CO_2_ from the first week could be used throughout the study because changes in δ^13^C due to incubation are relatively small (*53*) compared to changes induced by addition highly enriched substrates, as in this study. Soils were maintained at 45% WHC during the incubation to allow soils to be within optimal range for microbial activity. Addition of ^13^C-DOC to high C input microcosms was accomplished through a syringe to the microrhizon sampler to enable consistent labeling of the same soil area each week for 25 weeks. Upon DOC application, the area of soil wetted was observed and manually outlined with a marker onto both sides of the acrylic surface to precisely trace DOC application area. For low C input microcosms, custom needles, 140 mm in length (Hamilton Company, Reno, NV), were inserted to a different location within the microcosms each week to allow for diffuse ^13^C-DOC labeling across the entire volume of the microcosm. A consistent volume of ^13^C-DOC was added to low and high C input microcosms of the same plot, with a consistent scaling per g soil resulting in 1.4 to 1.6 ml of ^13^C-DOC added to each microcosm each week. Total DOC added throughout the incubation was scaled to g dry soil per microcosm and therefore varied on a microcosm basis between 24.5 and 27.6 mg C for low C input treatments and between 48.9 and 55.2 mg C for high C input treatments. Application rates were determined in reference to a previous ^13^C-labeling incubation work (*31*) and reflected in situ rhizodeposition from an agricultural cover crop (*54*).

### CO_2_ and δ^13^C-CO_2_ measurements

CO_2_ flux rates and δ^13^C of CO_2_ fluxes were quantified periodically throughout the incubation. On the basis of pilot studies with high frequency gas sampling from a subset of soils, we observed that the flux of CO_2_ from added ^13^C-DOC was low within the first hour after DOC addition and then increased rapidly to a peak 7 to 9 hours after DOC addition and subsequently declined. To describe the emission of ^13^C-DOC at both “phases” of emission: (i) “peak” and (ii) the low, steady state of emission we term “off-peak,” we sampled microcosms in a consistent pattern at 7-week intervals throughout the incubation (weeks 1, 7, 13, 19, and 25). After an initial gas sampling on the same day of soil wetting and packing into microcosms (week 0), microcosms were incubated for 1 week without any ^13^C-DOC addition. Hereafter, all gas sampling bouts consisted of (i) an initial off-peak gas sampling, followed within 4 min by ^13^C-DOC addition, (ii) a peak gas sampling, which was conducted 7 hours after initial gas sampling for weeks 1, 7, 13, and 19 and 1 hour after ^13^C-DOC addition in week 25, and (iii) a follow-up off peak gas sampling, 4 min prior to the ^13^C-DOC addition for the following week.

Although the exact peak rate of ^13^C-DOC emissions for each microcosm across the duration of the study could not be assessed, we chose 7 hours after ^13^C-DOC addition for the first four bouts of gas sampling as a timing that could be integrated into a workday and could also capture ^13^C-DOC flux behavior near peak emission rate. The interval between ^13^C-DOC addition and gas sampling was shortened to 1 hour on the last week of sampling under the intuition that time to peak ^13^C-DOC flux had shortened over the course of the incubation, possibly due to microbial adaptation to the supplied substrate. Because of the time-intensive nature of individual gas samplings, only the first three blocks (of three to five) from each site were selected for gas sampling throughout the study.

To conduct each gas sampling interval, an airtight, custom, acrylic chamber was used to sequentially enclose a microcosm in a closed-loop system with a Picarro G2101-*i* gas analyzer (Picarro Inc., Santa Clara, CA). The internal dimensions of this gas sampling chamber were 4 cm by 27 cm by 13.3 cm. For each gas sampling interval, the microcosm was kept in the sampling chamber for 240 s, while the soil in the microcosm, which was open to the chamber, respired. Of the 240-s sampling interval, 130 s was used to calculate both the CO_2_ flux rate and the δ^13^C of CO_2_ flux. After each sampling interval, the gas analyzer was allowed to flush built-up CO_2_ by sampling ambient air for 4 min. Concentrations of CO_2_ in ambient air at the initiation of gas sampling intervals did not exceed 500 ppm (parts per million). The proportion of CO_2_ flux from ^13^C-DOC versus existing SOC, $f_{A}$ , was calculated using a two-pool mixing model

$$f_{A}=\frac{\left(\delta_{S}-\delta_{B}\right)}{\left(\delta_{A}-\delta_{B}\right)}$$

where $\delta_{S}$ is the intercept of the Keeling plot for a given sampling point after ^13^C-DOC addition, $\delta_{B}$ is the average intercept of two Keeling plots from the first two gas sampling intervals, both of which occurred before any ^13^C-DOC had been applied, and $\delta_{A}$ is the δ^13^C of DOC, determined through EA-IRMS analysis on freeze-dried ^13^C-DOC.

### Destructive harvest of microcosms

After the 25th weekly addition of ^13^C-DOC, microcosms were allowed to incubate for a final week and the bout of gas sampling occurring over week 25 was completed before destructive harvest. Small losses of water were corrected for by bringing microcosms to 45% WHC. For low C input microcosms, all soil was immediately passed through an 8-mm sieved, and to minimize time spent sieving, a subsample was 2-mm sieved and kept fresh for assays described below. For high C input microcosms, customized templates for each microcosm were produced to help delineate concentration area of C application identified during ^13^C-DOC labeling. Soils labeled with ^13^C-DOC were gently separated from nonlabeled soils, and the entirety of labeled soils were 8-mm sieved and then 2-mm sieved. Soils sieved to 2 mm from low C input and high C input microcosms were handled in the same manner for the CUE (including microbial biomass) and SOC fractionation procedures described below.

### Microbial CUE

Within 48 hours after destructive harvest of microcosms, 2-mm sieved soils were assayed for microbial CUE using the ^18^O labeling method (*37*). This approach provides a substrate-independent assessment of microbial necromass production and CUE and involves microbial biomass measurements, ^18^O labeling incubation with respiration measurement, followed by DNA extraction and quantification.

#### Microbial biomass via chloroform fumigation

From each microcosm, ~5 g of fresh soil was weighed out twice, for fumigated and unfumigated aliquots (*55*). Unfumigated samples were immediately extracted with 25 ml of 0.5 M K_2_SO_4_ by shaking for 2 hours and then filtered through Whatman #42 filter papers. Fumigated samples were subjected to chloroform fumigation for 72 hours prior to the same extraction procedure with 0.5 M K_2_SO_4_. All K_2_SO_4_ extracts were acidified with three drops of phosphoric acid prior to freezing. Frozen extracts were subsequently freeze-dried prior to analysis via EA-IRMS (described above) for quantification of microbial biomass C and δ^13^C. Microbial biomass was divided by a correction factor of 0.45 (*55*).

#### ^18^O labeling and CUE respiration

From each microcosm, two aliquots of ~500 mg of fresh, 2-mm sieved soil were weighed into microcentrifuge tubes. Soil in one microcentrifuge tube was injected with a dilution of 97 at % ^18^O-enriched water, calculated to bring soil to 60% WHC and final soil water to ~20 ^18^O at %. The other microcentrifuge received an equivalent amount of natural abundance Milli-Q water. Immediately following addition of water (^18^O-enriched or natural abundance), each microcentrifuge tube was placed in a 20-ml serum bottle and sealed with aluminum crimp cap and rubber stopper. Every 10 samples, a blank serum vial was sealed for estimation of baseline CO_2_ concentrations. All samples were incubated in the dark for 72 hours at 25°C, at which point CO_2_ headspace concentrations of ^18^O-enriched vials were measured on an LI 7810 with a 5-ml injection, and all soil samples immediately frozen in liquid nitrogen and stored at −80°C until DNA extraction.

Soil DNA was extracted using the FastDNA SPIN Kit for Soil, with slight modifications from the manufacturer’s instructions: Centrifugation after bead-beating was extended to 15 min; the whole of the binding matrix suspension was transferred to the SPIN filter, and molecular biology grade water was used in the elution step. Given the low variability of ^18^O in natural abundance DNA controls, a randomized subset of 10% of control microcentrifuge tubes was analyzed to determine natural abundance of ^18^O in DNA. The DNA in extracts were quantified by fluorescence stain (Quant-iT PicoGreen dsDNA Reagent) and assessed using a microplate reader, with standards and samples in triplicate.^18^O concentrations in DNA were quantified by drying DNA extract at 60°C in silver capsules for TC/EA-IRMS analysis (UC Davis Stable Isotope Facility). As the ^18^O-CUE method assumes that microbes use only water-derived oxygen for their DNA synthesis, it may underestimate CUE [see (*56*) for further discussion].

Given information on microbial biomass (MBC), and information on respiration, which is based on the amount of CO_2_-C produced during the incubation, and information on growth, which is based on the amount ^18^O uptake in DNA total C uptake, CUE was calculated as

$$C_{\text{Uptake}}=C_{\text{Growth}}+C_{\text{Respiration}}$$

$$\mathrm{CUE}=\frac{C_{\text{Growth}}}{C_{\text{Growth}}+C_{\text{Respiration}}}$$

Given the assumption of steady-state conditions during the CUE incubation (i.e., growth = death), $C_{\text{Growth}}$ is interpreted as a rate of microbial necromass production, expressed as unit C per g dry soil hour^−1^. For further discussion of CUE assumptions and interpretation, see (*56*).

### Soil fractionation and formation efficiency of MAOC

SOC in baseline (preincubation) soils was assessed and reported in (*2*, *18*). The same size fractionation method was applied to incubated soils. Soils were separated by size into sand + POC (>53 μm) and silt + clay + MAOC (<53 μm) following (*57*). These fractions are hereafter referred to as “POC” and “MAOC,” respectively. Briefly, 5.75 to 6.25 g of 2-mm sieved bulk soil dried at 60°C was shaken for 18 hours with 12 glass beads in 30 ml of 0.5% sodium hexametaphosphate to disrupt all aggregates. The resulting soil slurry was rinsed with DI water over a 53-μm sieve to isolate POC and remove glass beads. Soil solution passing through the sieve was deemed MAOC. Both the POC and MAOC fractions were dried at 60°C until reaching constant mass. Recoveries of the initial soil masses in the summed fractions were between 96 and 105% for all samples, with a mean recovery of 101.7%. Soils and fractions containing carbonates (identified by effervescence after addition of 5% HCl, 36 samples) were treated to remove inorganic carbon via HCl fumigation (*58*). SOC, MAOC, and POC were ground using a mortar and pestle before running on an EA-IRMS (Elemental Analyzer–Isotope Ratio Mass Spectrometry; vario ISOTOPE cube, Elementar, Langenselbold, Germany). The average recovery of SOC in POC and MAOC fractions was 89%.

A two-pool mixing model was used to estimate the proportion of C in SOC, POC, and MAOC derived from the added ^13^C-DOC

$$f_{A}=\frac{\left(\delta^{13}C\;\text{incubated sample}-\delta^{13}C\;\text{baseline sample}\right)}{\left(\delta^{13}C\text{-}\mathrm{DOC}-\delta^{13}C\;\text{baseline sample}\right)}$$

where $f_{A}$ is the fraction C from ^13^C-DOC in the C pool, $\delta^{13}C$ incubated sample is the δ value of that C pool after incubation, $\delta^{13}C\;\text{baseline sample}$ is the δ value of that C pool before incubation, and $\delta^{13}C\text{-}\mathrm{DOC}$ is the δ value of the added DOC. Each $f_{A}$ was multiplied by the g C kg soil^−1^ on the incubation fraction to arrive at a quantity of DOC-derived C per mass soil. The recovery of the ^13^C-DOC in the unlabeled portion of the high C input microcosms was, on average, 4% of the applied label. Incorporating the unlabeled area of high C input treatments to calculations of MAOC formation efficiency increased MAOC formation efficiency from 22.6 to 25.6% and did not change comparison of C input treatment effects. We therefore report formation efficiencies from the labeled area of high C input treatments. Formation efficiency of MAOC was calculated as

$$\mathrm{ME}=f_{A}\;\text{MAOC}\left(\frac{1}{\mathrm{DOC}}\right)$$

where ME is MAOC formation efficiency, $f_{A}$ is the fraction of MAOC from ^13^C-DOC (defined above), MAOC is the total mass of labeled MAOC per microcosm, and DOC is the total mass of ^13^C-DOC applied per microcosm.

### Estimation of peak and off-peak ^13^C-DOC respiration

Total respiration of ^13^C-DOC during off-peak respiration was estimated by integrating the area under the gas curve, using the 10 off-peak measurements for each microcosm. We first fit Michaelis-Menten curves to describe off-peak respiration. Of 180 microcosms gas sampled, 167 could be fitted with Michaelis-Menten functions; another 6 microcosms were suitably fit with linear regression models based on visual inspection. Gas sampling series from 7 microcosms were deemed of insufficient quality for integration and removed from subsequent analyses, leaving 173 microcosms for gas sampling analyses. Given the estimate of total off-peak respiration per microcosm, a known quantity of ^13^C-DOC added, and an estimate of ^13^C-DOC recovered in soil, respiratory losses of ^13^C-DOC during peak fluxes were calculated as the difference between ^13^C-DOC added and ^13^C-DOC otherwise accounted for (soil + off-peak respiration).

### Estimation of MAOC saturation and saturation deficit

Following previous works (*2*, *15*), we estimated the theoretical C saturation of the MAOC fraction in our soils. Briefly, to derive the boundary line from the data in (*15*), we used the observations for g MAOC kg soil^−1^ as a function of silt + clay, first identifying observations in the 95th percentile or higher MAOC concentration in each of 10 segments of silt + clay content (i.e., the first segment of observations with <10% silt + clay, the next segment of observations with greater than or equal to 10% silt + clay and <20%, etc.). Using simple linear regression of these boundary points with the intercept forced through zero, on the assumption that soils without silt + clay have no MAOC, we estimated the boundary line slope of MAOC ~ silt + clay to be 0.7323. To calculate theoretical MAOC saturation in g MAOC kg soil^−1^ for all soils, we multiplied silt + clay by 0.7323 (our boundary line slope). We calculated the absolute MAOC saturation deficit by subtracting observed g MAOC kg soil^−1^ from theoretical g MAOC kg soil^−1^ at saturation.

### Statistical analyses

We used multilevel, two-sided Pearson’s correlations with 95% confidence levels to assess linear relationships between MAOC formation (mg ^13^C-DOC-derived MAOC kg soil^−1^) and soil physicochemical properties. Before all analyses, a uniform outlier cleaning procedure was applied. Individual data points were flagged as outliers and removed from subsequent analyses if they fell below the 25th percentile minus 1.5 times the IQR or above the 75th percentile plus 1.5 times the IQR.

We used structural equation modeling to assess inter-related drivers of new MAOC formation in an initial full model and then in a simplified model. In both cases, we avoided including soil silt + clay contents and saturation deficit in the same model due to their high correlation (*r* = 0.9). To assess whether relationships between variables differed between high and low C input treatments, we standardized variables included in the model and then compared multilevel SEM, where high and low C input data were each assigned a level. An unconstrained model, where regression coefficients were freely estimated, was first compared to a constrained model, where these coefficients were set to be equal across groups. This comparison, assessed through a chi-square difference test, aimed to determine whether the regression relationships differ significantly between groups. The resulting *P* values of >0.5 for the analysis of variance (ANOVA) of chi-square values indicated no significant difference between the constrained and unconstrained models, suggesting that the relationships among drivers of MAOC formation were consistent across C input levels. All structural equation models met model fit indices (*59*) and were created using global estimation in the lavaan package in R (*60*).

We tested for differences in the mean MAOC formation efficiency, % ^13^C-DOC respired during peak, microbial CUE, and microbial growth between high and low C input treatments using Wilcoxon rank sum tests. A nonparametric test, like Wilcoxon rank sum, was preferred over a *t* test as Shapiro-Wilk tests and Q-Q plots established that all pairs of comparisons had at least one set of non-normally distributed data.
