# Supplementary material for: Soil carbon formation is promoted by saturation deficit and existing mineral-associated carbon, not by microbial carbon-use efficiency
Source: Sci Adv. 2025 Jun 13;11(24):eadv9482. doi: 10.1126/sciadv.adv9482 (PMC13109956; doi:10.1126/sciadv.adv9482)
Supplement: Supplementary file 1 — Figs. S1 to S9 Tables S1 and S2 [file sciadv.adv9482_sm.pdf]

Science Advances
AAAS

Supplementary Materials for

Soil carbon formation is promoted by saturation deficit and existing mineral-associated carbon, not by microbial carbon-use efficiency

Alison E. King and Noah W. Sokol

Corresponding author: Alison E. King, alison.king@maine.edu

Sci. Adv. **11**, eadv9482 (2025)
DOI: 10.1126/sciadv.adv9482

This PDF file includes:

Figs. S1 to S9
Tables S1 and S2

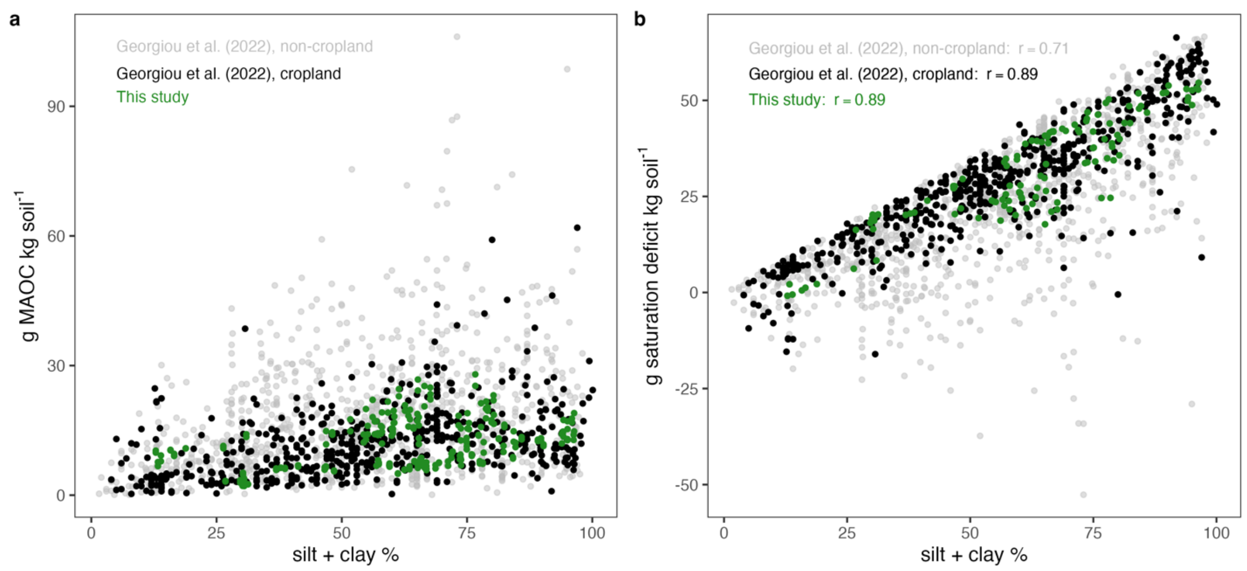

Fig. S1. Soils in this study, vis-à-vis a global dataset. of a) Influence of silt + clay % on mineral-associated organic carbon (MAOC) for dataset of Georgiou et al. (2022) (*15*) and this study. Georgiou et al. (2022) points are shown for non-cropland (gray) and cropland (black); points for this study, which represent cropland soils, are shown in green. b) Relationship between silt + clay % and saturation deficit (g kg soil^{-1}) for Georgiou et al. (2022) non-cropland (gray), Georgiou et al. (2022) cropland (black) and this study (green). Pearson's correlation coefficients (r) shown for each group. Saturation deficit is calculated as the difference between MAOC concentration at saturation (using global slope of silt + clay% * 0.73) and the observed MAOC concentration.

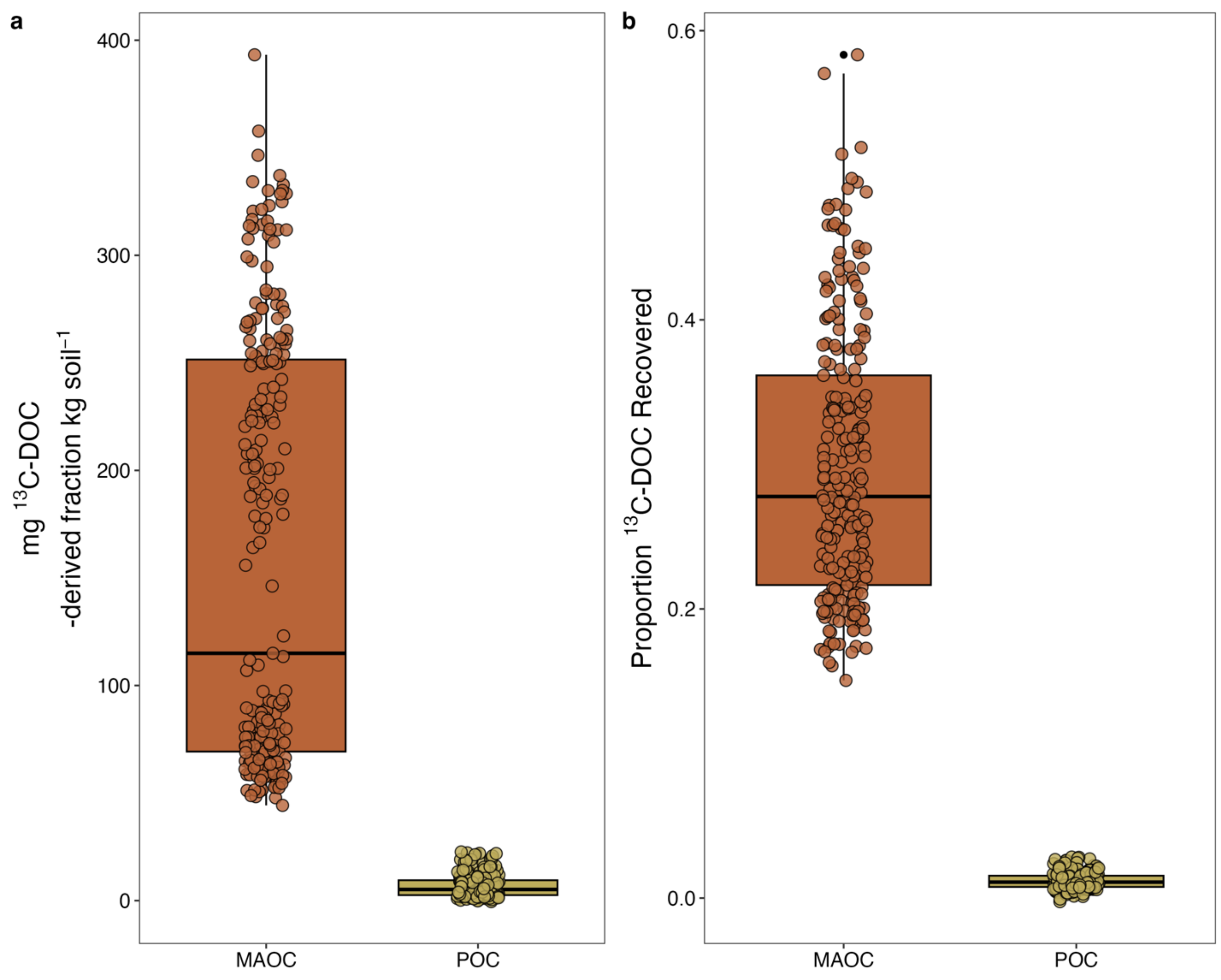

Fig. S2. Recovery of a dissolved organic carbon (DOC) label in mineral-associated organic carbon (MAOC) and particulate organic carbon (POC). a) Formation of MAOC and POC from a ^{13}C-DOC label. Average MAOC formation is 161 mg MAOC kg soil^{-1} (standard deviation, SD = 97.8); average POC formation is 6.8 mg POC kg soil^{-1} (SD = 5.4). b) Proportion of ^{13}C-DOC label recovered in MAOC and POC. Average label recovery in MAOC is 29.8% (SD = 9.4%); average label recovery in POC is 1.2% (SD = 0.5%).

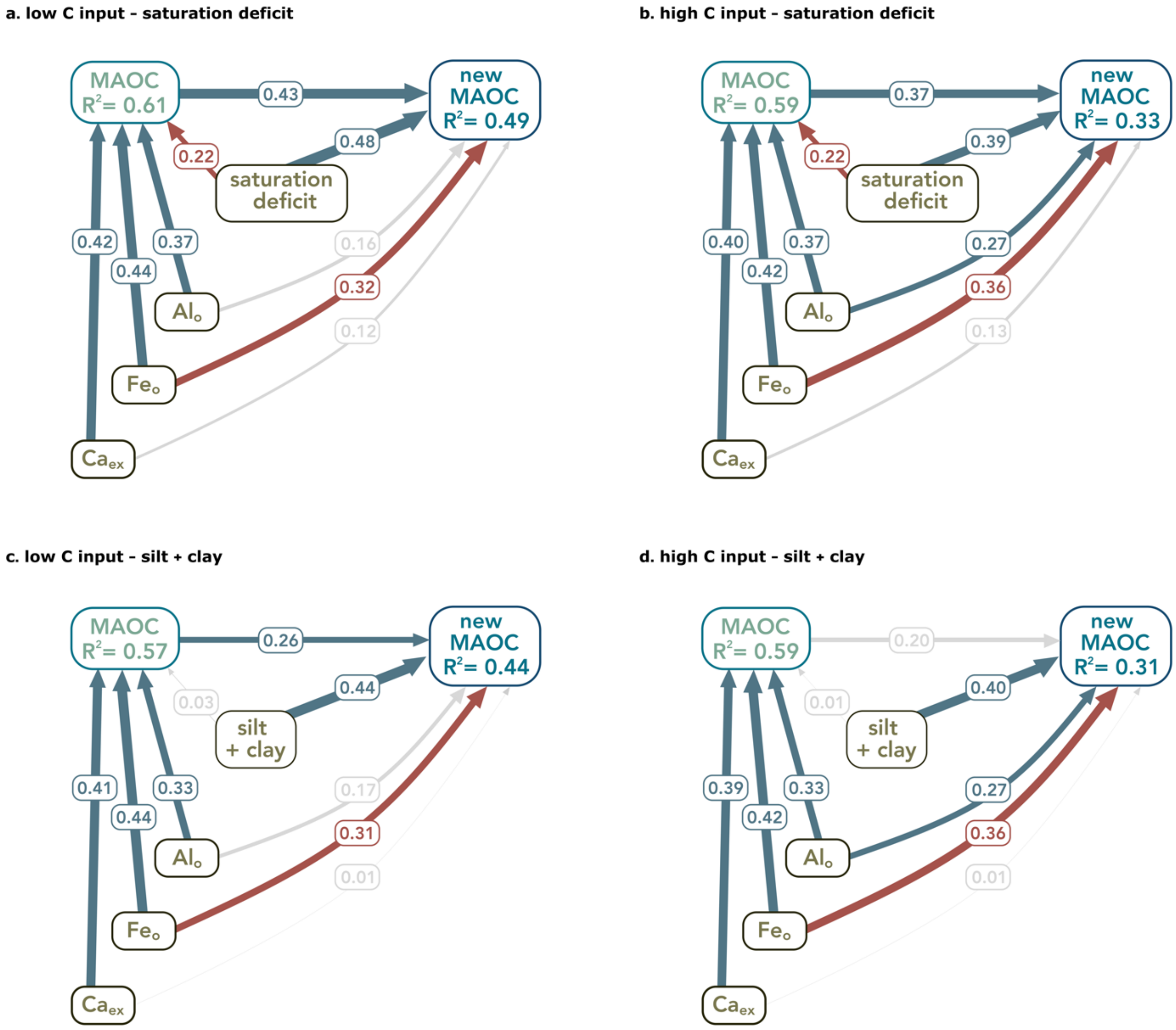

Fig. S3. Multi-group SEM at both levels of C input. Either saturation deficit (a, b) or silt + clay (c, d) represent a mineralogical influence on both existing mineral-associated organic carbon (MAOC) and formation of new MAOC. Blue paths represent significant positive relationships ($p < 0.05$); red paths represent significant negative relationships ($p < 0.05$); gray path represents non-significant relationships. Both models have comparative fit index (CFI) > 0.95 and root mean square error of approximation (RMSEA) < 0.08. Path coefficients are standardized.

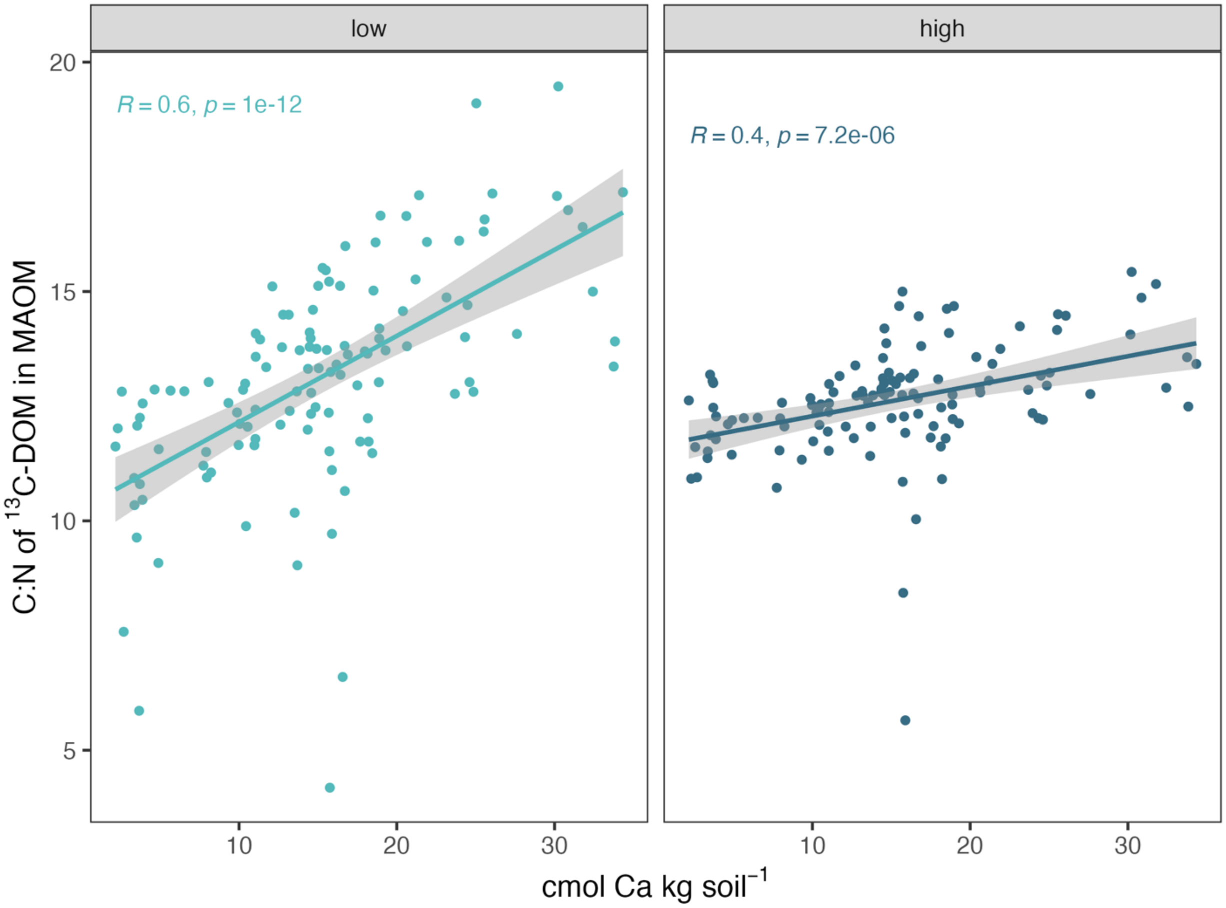

Fig. S4. Relationship between C:N ratio of ^{13}C-DOC-derived mineral-associated organic matter (MAOM) and exchangeable calcium (Ca) across C input treatments.

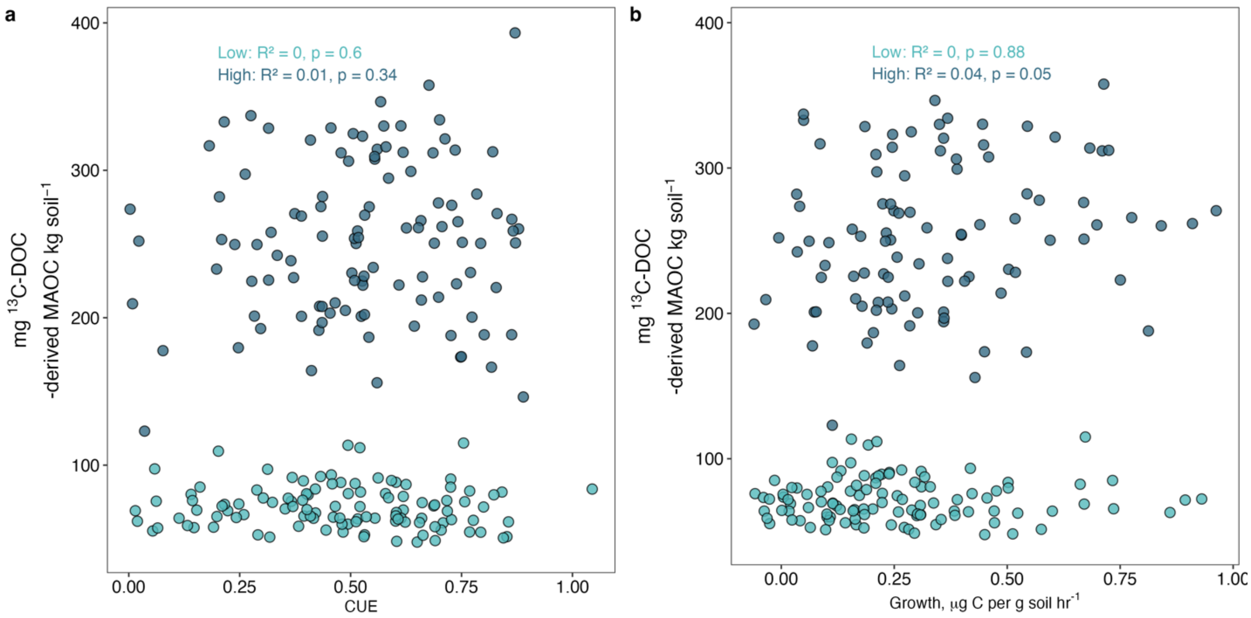

Fig. S5. **Formation of mineral-associated organic carbon (MAOC) in relation to microbial traits and C input treatment.** a) formation of MAOC in both C input treatments as a function of CUE and b) formation of MAOC in both C input treatments as a function of microbial growth rate.

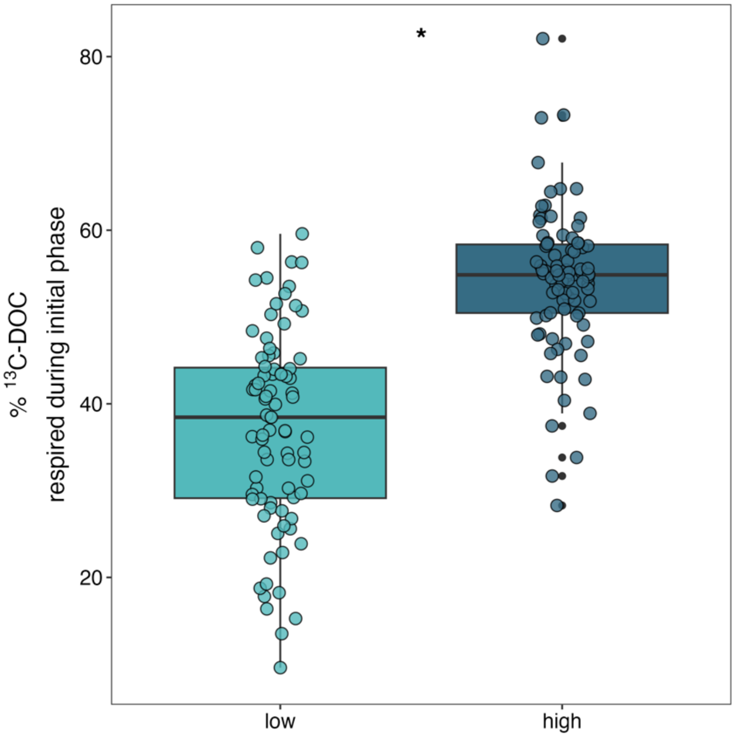

Fig. S6. Percent ^{13}C-dissolved organic carbon (DOC) respired during initial (peak) respiratory flux between C input treatments. Percent respired during initial phase is calculated as the difference of (100% [of ^{13}C-DOC applied per microcosm] – (% of applied ^{13}C-DOC recovered in microcosm + % of applied ^{13}C-DOC respired in offpeak phase)). The % of applied ^{13}C-DOC respired in the offpeak phase was calculated from the integral of offpeak measurements during the incubation (see Figure S7 for conceptual diagram).

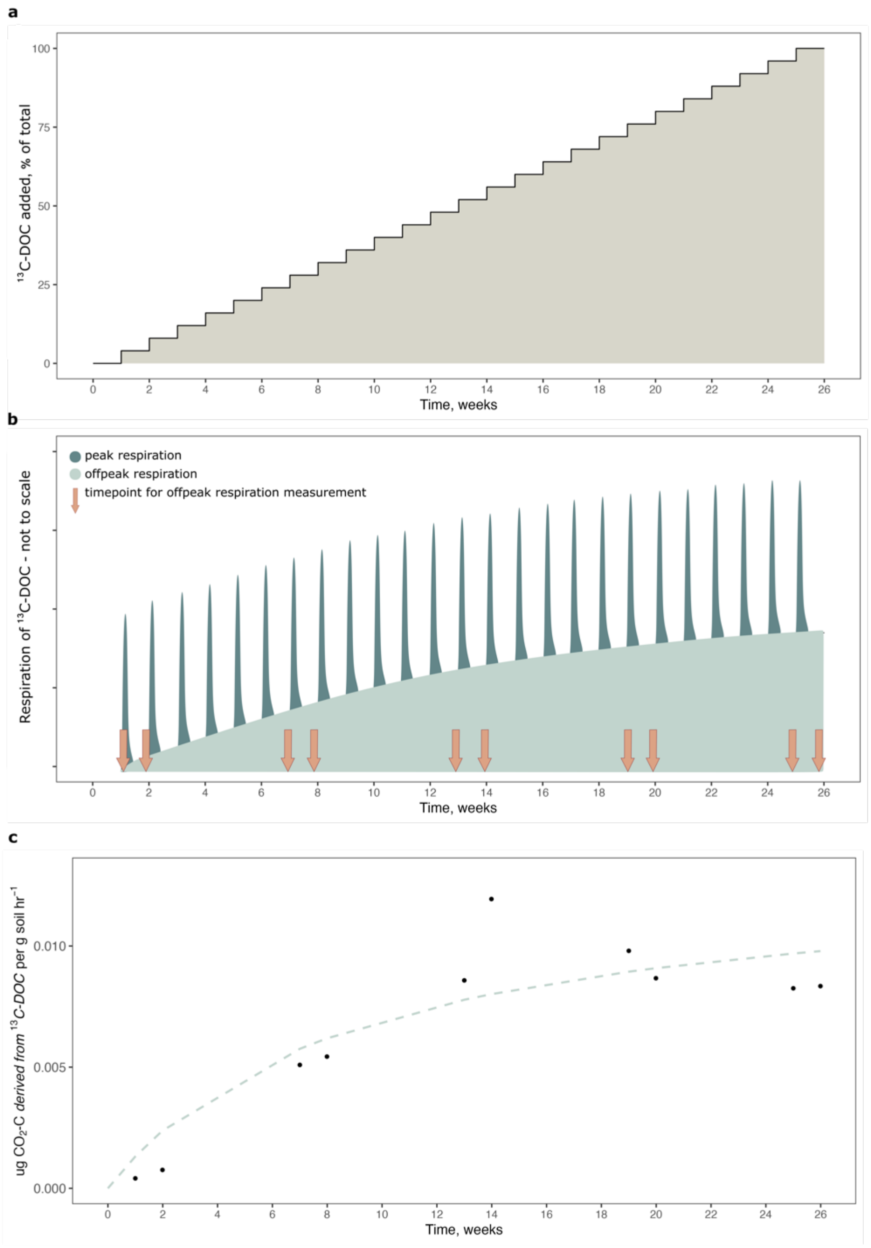

Fig. S7. **Additions of ^{13}C-dissolved organic carbon (DOC) and respiration dynamics throughout the 6-month incubation**. a) Cumulative weekly additions of ^{13}C-DOC; b) conceptual diagram of ^{13}C-DOC respired during initial phase (peak) and off-peak phase, also showing timing of off-peak gas sampling measurements; c) data from microcosm 59, an example of an off-peak respiration curve. The area under this curve is integrated to estimate total off-peak respiration during the incubation.

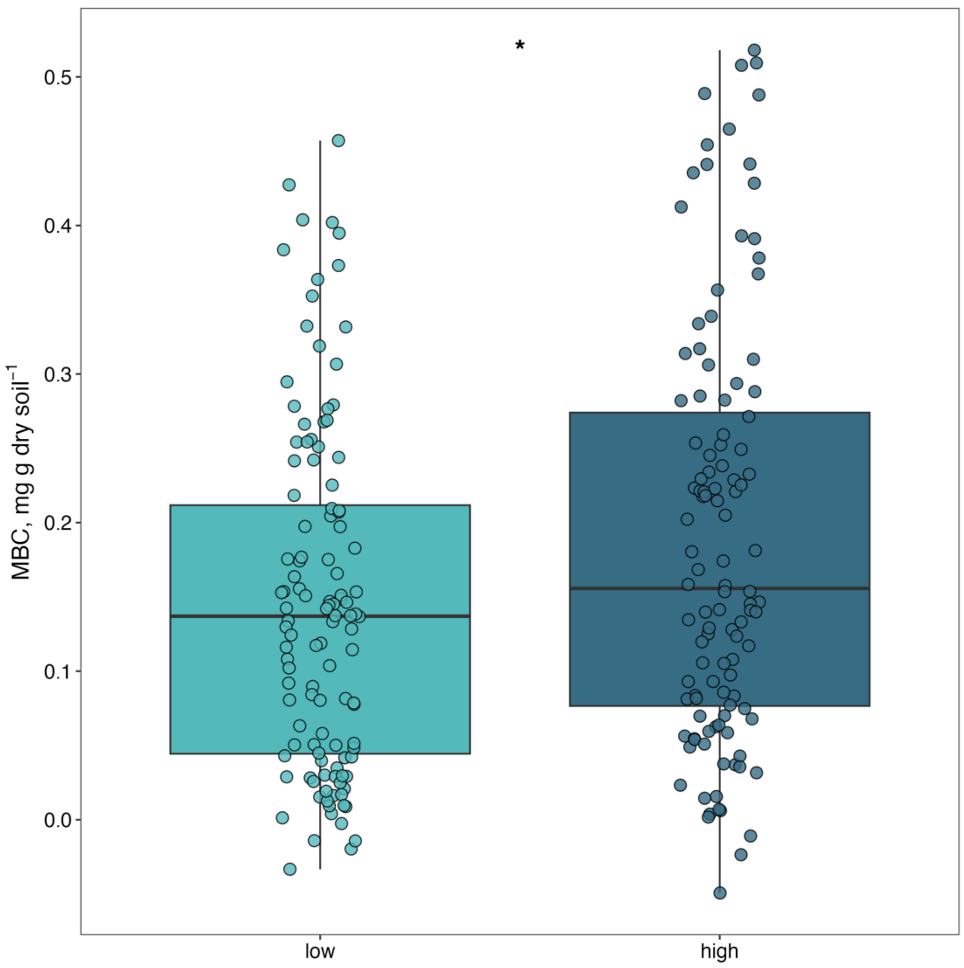

Fig. S8. Microbial biomass carbon (MBC) across low and high C input treatments. Difference between treatments indicated with asterisk (p = 0.023, Wilcoxon rank-sum test).

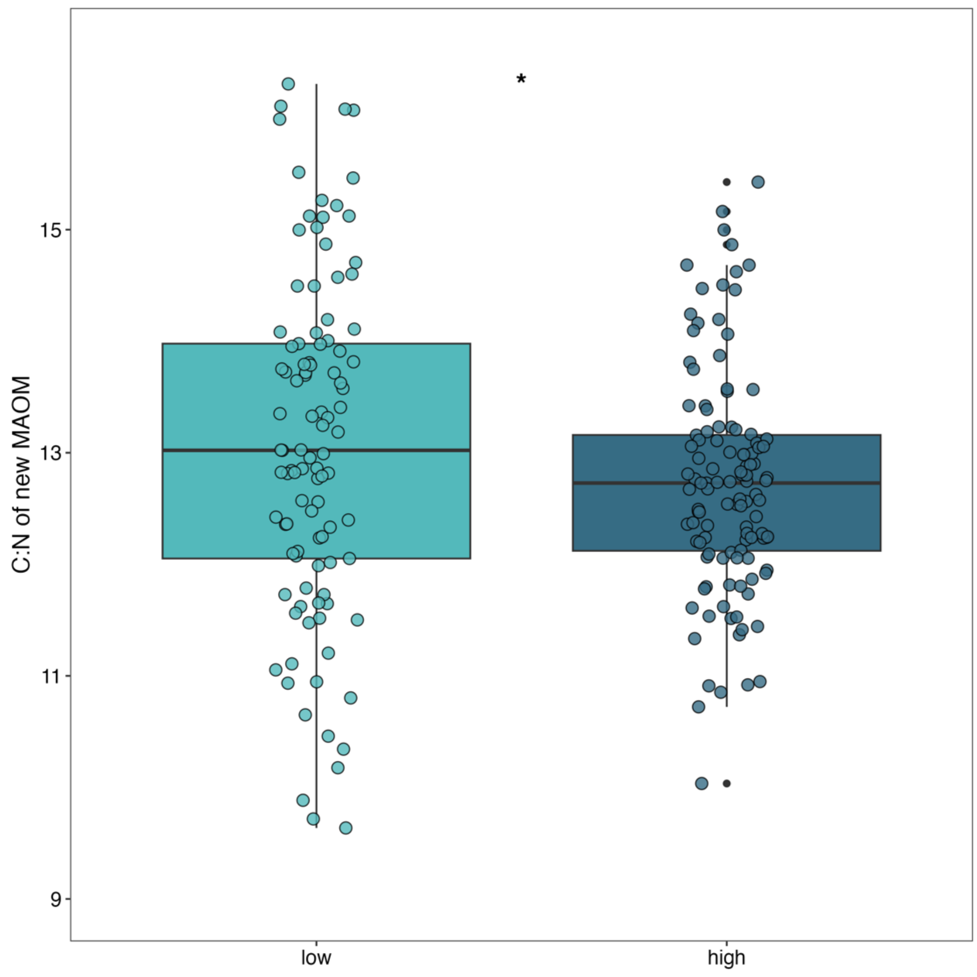

Fig. S9. Ratio of carbon to nitrogen (C:N) of newly formed mineral-associated organic matter (MAOM) across low and high C input treatments. Difference between treatments indicated with asterisk ($p = 0.047$, Wilcoxon rank-sum test).

Table S1. Summary of soil properties for 118 incubated soils. Silt + clay and sand are reported in percent. Soil organic carbon (SOC), mineral-associated organic carbon (MAOC), and saturation deficit (Sat. deficit) are reported in g kg soil^{-1}; Ca_{ex} = exchangeable calcium (cmol kg soil^{-1}) Al_O = oxalate-extractable aluminum (g kg soil^{-1}); Fe_O = oxalate-extractable iron (g kg soil^{-1}). Full dataset available in a Figshare repository (https://doi.org/10.6084/m9.figshare.25035818).

Soil property	min.	mean	max.
Silt + clay	12.9	62.3	96.4
Sand	3.6	37.7	87.1
SOC	2.9	15.8	38.6
MAOC	2.1	12.1	28
Sat. deficit	−0.9	31.9	54.6
Ca_{ex}	2.2	14.5	30.9
Al_O	0.7	1.3	1.9
Fe_O	0.1	1.6	4.3
pH	5.3	6.5	8.2

Table S2. Model output for 9 multiple regression models predicting the formation of new mineral-associated organic carbon (MAOC), showing that inclusion of both saturation deficit (Sat Deficit) and existing MAOC improves model performance compared to inclusion of either one alone. All predictor variables and MAOC formation are standardized within carbon input level, for interpretability of coefficients. Al_O = oxalate-extractable aluminum; Fe_O = oxalate-extractable iron; Ca_{ex} = exchangeable calcium. R^2 values are all adjusted R^2. AIC = Akaike Information Criterion; BIC = Bayesian Information Criterion.

		**low C input**			**high C input**			**combined**	
**model**	predictor	coefficient	p-value	predictor	coefficient	p-value	predictor	coefficient	p-value
MAOC + Al_O + Fe_O + Ca_{ex}	(Intercept)	0.11	0.0492	(Intercept)	0.29	0	(Intercept)	0.2	0
	Al_O	0.33	0.0024	Al_O	0.39	4.00E-04	Al_O	0.35	0
	Ca_{ex}	0.2	0.0189	Ca_{ex}	-0.03	0.7354	Ca_{ex}	0.08	0.2232
	Fe_O	-0.18	0.0707	Fe_O	-0.23	0.0252	Fe_O	-0.21	0.0033
	MAOC	0.2	0.1106	MAOC	0.16	0.1536	MAOC	0.21	0.0148
	**R^2: 0.27, AIC: -69.96, BIC: -53.92**			**R^2: 0.17, AIC: -62.24, BIC: -46.20**			**R^2: 0.21, AIC: -125.08, BIC: -104.88**		
Sat Deficit + Al_O + Fe_O + Ca_{ex}	(Intercept)	-0.04	0.4685	(Intercept)	0.17	0.0056	(Intercept)	0.07	0.1303
	Al_O	0.31	8.00E-04	Al_O	0.39	1.00E-04	Al_O	0.35	0
	Ca_{ex}	0.25	3.00E-04	Ca_{ex}	0.01	0.8649	Ca_{ex}	0.13	0.0121
	Fe_O	-0.11	0.1461	Fe_O	-0.17	0.0466	Fe_O	-0.14	0.0188
	Sat Deficit	0.31	0	Sat Deficit	0.24	7.00E-04	Sat Deficit	0.28	0
	**R^2: 0.39, AIC:-89.48, BIC:-73.44**			**R^2: 0.24, AIC: -72.34, BIC: -56.30**			**R^2: 0.29, AIC: -149.10, BIC: -128.91**		
Sat Deficit + MAOC + Al_O + Fe_O + Ca_{ex}	(Intercept)	-0.04	0.4706	(Intercept)	0.17	0.004	(Intercept)	0.07	0.1018
	Al_O	0.15	0.1058	Al_O	0.26	0.0141	Al_O	0.2	0.0077
	Ca_{ex}	0.1	0.1962	Ca_{ex}	-0.11	0.1911	Ca_{ex}	-0.01	0.7961
	Fe_O	-0.28	0.0016	Fe_O	-0.3	0.0018	Fe_O	-0.3	0
	MAOC	0.42	3.00E-04	MAOC	0.31	0.005	MAOC	0.39	0
	Sat Deficit	0.39	0	Sat Deficit	0.31	0	Sat Deficit	0.35	0
	**R^2: 0.46, AIC: -101.42, BIC: -82.71**			**R^2: 0.29, AIC: -78.74, BIC: -60.03**			**R^2: 0.36, AIC: -170.28, BIC: -146.72**		
